# An Unusual Presentation of Prostatic Abscess due to Actinomyces turicensis and Peptostreptococcus

**DOI:** 10.7759/cureus.8665

**Published:** 2020-06-17

**Authors:** Anastasia Barnes, Avneet Kaur, Michael Augenbraun

**Affiliations:** 1 Infectious Diseases, State University of New York Downstate Medical Center, Brooklyn, USA

**Keywords:** actinomyces, peptostreptococcus, prostatic abscess

## Abstract

Actinomyces is a rare cause of prostatic abscess. Most reported cases of abdominal and pelvic actinomycosis are a consequence of invasive procedures or abdominal infections. In this report, we present a previously healthy 53-year-old man with inguinal pain, fever and dysuria who was found to have a prostate abscess, secondary to Actinomyces turicensis and Peptostreptococcus, which was removed via transurethral resection of the prostate. This case was complicated by Peptostreptococcus bacteremia and a facial abscess. The patient was treated with antibiotics and ultimately made a full recovery.

## Introduction

Prostatic abscesses are uncommon but potentially serious disorders. They may occur as sequelae of bacterial prostatitis or urinary reflux, or may result from hematogenous spread from other organs [1]. They are difficult to diagnose, given that patients present with nonspecific symptoms of fever, dysuria and acute urinary retention [2]. They are more common in immunocompromised patients (e.g. HIV, diabetes) [1]. While in the past Neisseria gonorrhoeae caused 75% of cases of prostatic abscess, with the current availability of antibiotics, the most common offending organism is Escherichia coli (60%-80%) [2]. This report of a prostatic abscess is particularly interesting as the culture grew Actinomyces turicensis and Peptostreptococcus.

## Case presentation

A 53-year-old man presented with a one-week history of left inguinal pain, subjective fever and chills, and two episodes of vomiting. Two days prior to presentation, he also developed incomplete bladder emptying and dysuria. The patient had no significant past medical or surgical history. There was no history of prior lower urinary tract symptoms, genitourinary instrumentation or catheterization, or of renal stones. There was a history of a gunshot wound to right posterior chest wall 35 years previously, with a retained bullet.

**Figure 1:**
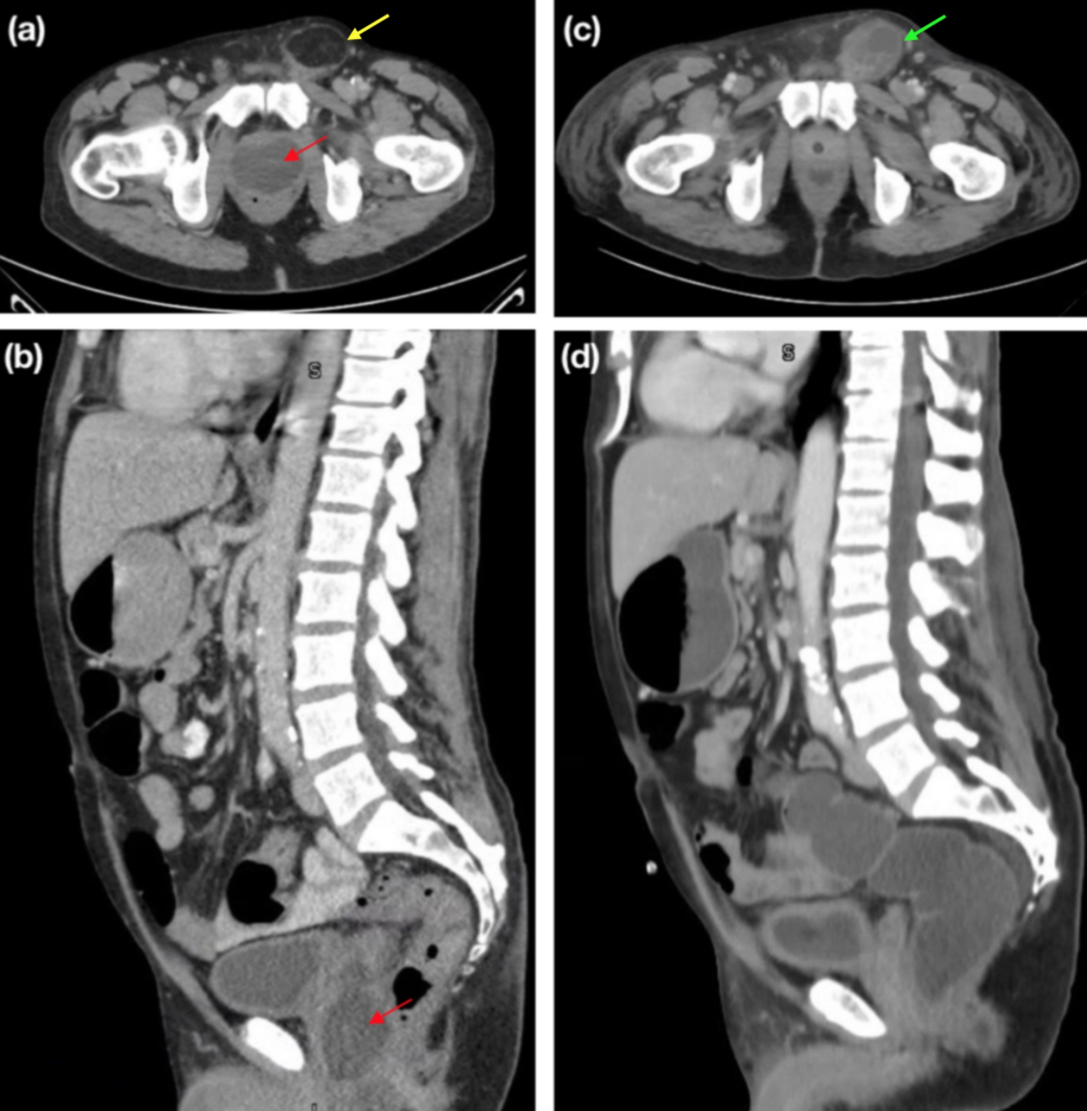
Prostatic Abscess (a, b) Cystic lesion within the prostate, approximately 6 x 2.7 cm (red arrows), and incidental inguinal hernia, approximately 5 x 2.5 cm (yellow arrow). (c, d) Interval resolution of cystic lesion within the prostate and development of a multiseptated cystic lesion in the left inguinal canal (green arrow).

In the emergency department, the patient had a temperature of 102.5ºF, a heart rate of 120 beats/minute and leukocyte count of 29.5 x 10^9^/L (neutrophil count: 25.5 × 10^9^/L). He was unable to tolerate a digital rectal exam due to pain. Urinalysis showed large leukocyte esterase, blood and moderate bacteria. Bladder sonography revealed a postvoid residual of 60 cc. CT of the abdomen and pelvis revealed a large 6 x 2.7 cm prostate abscess tracking into the seminal vesicles bilaterally and an incidental 5 x 2.5 cm left inguinal hernia (Figures 1a, 1b).

The patient was treated with vancomycin and piperacillin-tazobactam and underwent transurethral resection of the abscess, with the placement of Foley and suprapubic catheters. However, his condition was complicated by septic shock requiring mechanical ventilation and vasopressors (norepinephrine infusion) and transfer to the medical ICU. He stabilized two days later; he was successfully extubated and transferred to the urology service.

A repeat CT was performed which showed resolution of the prostatic abscess and a new left inguinal cord abscess (Figures 1c, 1d), which required left groin exploration and drainage of a necrotic hernia sac on the fifth day of hospitalization. At that time, the patient also developed a right facial abscess in the mandibular region requiring incision and drainage by otolaryngology. On the sixth day of hospitalization, he was transferred to a medical unit and clinically improved. The prostate abscess culture grew Actinomyces turicensis and Peptostreptococcus; blood cultures and the facial abscess culture also grew Peptostreptococcus. In consultation with the infectious diseases service, antibiotics were switched to ampicillin/sulbactam. The patient’s Foley catheter was removed successfully with no immediate complications. A few days later, the patient was discharged with a peripherally inserted central catheter (PICC) line to complete IV ceftriaxone for two weeks followed by PO amoxicillin-clavulanic acid for six months. One week after the discharge, the patient was seen in the urology clinic and had no complaints other than occasional mild urinary incontinence. His suprapubic catheter was successfully removed at that time.

## Discussion

Actinomyces turicensis and Peptostreptococcus are especially rare causes of prostatic abscess. Actinomyces is a genus that consists of several species of gram-positive anaerobic bacilli [3]. These species exist as normal flora of the mouth, pharynx, esophagus and genitourinary tract. They may rarely cause infection in Western countries, particularly after disturbance of mucosal barriers (e.g. trauma, surgical procedures) [3]. Per Groeneveld et al., who report a case of Actinomyces neuii prostatic abscess in a patient with recurrent urinary tract infections, only a handful of Actinomyces prostatic soft tissue infections exist in the literature [4-8]. Diabetes mellitus was a predisposing factor in one of these cases [8].

Over the past few decades, several new species of Actinomyces have been discovered. Actinomyces turicensis was first described in 1995 [9]. This organism has been reported to cause a variety of genital infections, including balanitis, urethritis, penile abscess and prostatitis, but not yet prostatic abscess [10].

Actinomyces turicensis infections may occur with concomitant organisms including fellow anaerobes, such as Peptostreptococcus [10]. As gram-positive anaerobic cocci, Peptostreptococci are also normal inhabitants of the skin, gastrointestinal tract, mouth and upper respiratory tract [11]. They may cause abscesses of skin or soft tissue and a variety of infections (e.g. periodontitis, aspiration pneumonia, intra-abdominal) [11]. They are a common cause of anaerobic bacteremia [12,13]. They may also cause genitourinary tract infections, including chronic prostatitis, but to our knowledge have not yet been reported to cause an acute prostatic abscess [11,14,15].

The role of anaerobes in precipitating prostatitis, and thus prostatic abscess, is likely underestimated, as they are not routinely cultured in urine [15]. This is due to the fact that bacterial prostatitis is usually due to gram-negative aerobes (e.g. Escherichia coli, Klebsiella, Pseudomonas) [15]. However, anaerobes should also be considered as causative organisms, especially in chronic cases [15]. In a study of 50 patients with chronic prostatitis resistant to quinolone therapy, anaerobic bacteria were cultured from samples of urethral discharge and prostatic fluid from 24 of the patients [16]. Of note, Peptostreptococcus was the most common anaerobe isolated [16]. The authors note that failure to use anaerobic culture methods, which also require more stringent conditions such as a longer incubation period, may contribute to the misdiagnosis of prostatitis as non-bacterial [16].

There are no standard guidelines for the management of prostatic abscess given that it is a rare condition; however, in general, treatment should include parenteral broad-spectrum antibiotic administration and drainage of the abscess [1]. Abdelmoteleb et al. recommend that large abscesses (>1 cm) undergo transrectal ultrasound drainage and antibiotic therapy, followed by further imaging and surgical intervention if the patient fails to improve. Of note, our patient could not tolerate a digital rectal exam and thus was not a candidate for less-invasive ultrasound drainage [1]. However, there are also downsides to this approach; Jang et al. found that needle aspiration was associated with abscess recurrence, and that transurethral resection of the prostate had a more favorable outcome in terms of length of hospitalization [2].

## Conclusions

In this report, we describe a prostatic abscess secondary to Actinomyces and Peptostreptococcus complicated by sepsis in a previously healthy middle-aged man with no identifiable predisposing factors. The original source of the abscess is unknown, but the incidental hernia discovered on initial imaging and development of an inguinal cord abscess possibly implicates the gastrointestinal tract. Additionally, hematogenous spread of Peptostreptococcus to the face may explain subsequent formation of a facial abscess. The patient made a recovery to near baseline after transurethral resection of the prostate, hernia surgery, incision and drainage of the facial abscess, and antibiotic therapy.
